# Clinical and biological factors associated with the presence of peripheral neuropathy in patients with rheumatoid arthritis

**DOI:** 10.3389/fneur.2026.1755880

**Published:** 2026-04-21

**Authors:** Yannick Fogoum Fogang, Bleriol Fondjo Azemkeu, Claudine Sen Henriette Ngomtcho, Fernando Kemta Lekpa, Michel Noubom

**Affiliations:** Department of Neurology, Faculty of Medicine and Pharmaceutical Sciences, University of Dschang, Dschang, Cameroon

**Keywords:** associated factors, neuropathy, peripheral neuropathies, rheumatoid arthritis, rheumatoid factor (RF)

## Abstract

**Objective:**

Immune dysfunction in rheumatoid arthritis (RA) is a contributing factor to the development of peripheral neuropathy (PN). The objective of our study was to investigate the biological and clinical factors associated with PN in patients with RA.

**Materials and methods:**

We conducted a retro-prospective cross-sectional study. A total of 63 patients with RA were included. They were divided into two groups, 18 with PN and 45 without PN. Participants with PN were those with a pathological electroneuromyogram (ENMG) with or without signs and symptoms of PN. Blood samples were taken for the measurement of rheumatoid factor (RF) and C-reactive protein (CRP). The concentration of anti-citrullinated peptide antibodies (ACPA) were collected from patient records. The significance threshold was set at a *p*-value <0.05.

**Results:**

The majority of participants, 82.5% were female. The mean age was 51.86 ± 13.07 years. PN was present in 28.6% of the participants. RF and ACPA were positive in 71.4 and 77.8% of the participants, respectively. Severely active RA was significantly associated with the presence of PN (*p* < 0.001, OR = 55.13). RF concentrations were significantly higher in patients with PN. The area under the ROC curve for RF concentration in predicting PN in patients with RA was 0.7 (AUC = 0.7), patients with an RF > 169.10 IU/mL had a significant risk of presenting PN (*p* = 0.003, OR = 8.20).

**Conclusion:**

Severe rheumatoid arthritis is associated with PN. Inflammatory markers may play a key role in the pathogenesis and may provide valuable guidance for the early diagnosis of PN in RA.

## 1 Introduction

Rheumatoid arthritis (RA) is an immune pathology that develops in flares and primarily attacks the joints. According to ACR/EULAR (American College of Rheumatology/European League Against Rheumatism), the criteria for the biological diagnosis of RA include inflammatory biomarkers such as rheumatoid factor (RF), anti-citrullinated peptide antibodies (ACPA), C-reactive protein (CRP) and erythrocyte sedimentation rate (ESR) (1). The inflammatory imbalance in this pathology gradually leads to the destruction of bone and cartilage, and often causes the appearance of extra-articular manifestations during the disease (2, 3).

Peripheral neuropathy (PN), one of these extra-articular complications, occurs in 0.5 to 85% of patients with RA and presents as multiple mononeuropathy (11%), focal mononeuropathy (carpal tunnel: 18%), pure sensory (45%), or mixed neuropathy (40%) (4). Peripheral nerve involvement can manifest with a wide variety of symptoms, such as neuropathic pain, paresthesias which can mimic and overlap with those of arthritis. It is difficult in clinical practice to distinguish the symptoms of peripheral neuropathy from those of arthritis (5). The causes of peripheral neuropathies in RA are numerous and include: vasculitis; neurotoxicity of drugs used for the treatment of RA, infections, vitamin B deficiencies, and rarely amyloidosis (6). They are length-dependent or no length dependent, and sensory involvement generally occurs before motor involvement (7–9).

Some studies have shown the involvement of immune dysregulation in the development of peripheral neuropathies. Different pathological mechanisms are involved such as: T-cell or induced autoantibody attack against myelin, ischemic processes or inhibition of axon signaling. The clinical presentation spectrum seems to be heterogenous, presenting as axonal, symmetric sensory polyneuropathy or as mononeuropathy simplex or multiplex or even as a conduction block (10). The electroneuromyogram (ENMG) examination is normal in some cases of sensory peripheral neuropathies because Aδ and C fibers (small-fiber neuropathy) are not explored by the examination and require additional tests (10, 11). Almost 65% of patients may present with subclinical peripheral neuropathy (7). Thus, diagnosing peripheral neuropathy is not always easy in RA because symptoms of peripheral neuropathy share similarities with those of RA. Furthermore, diagnosis of peripheral neuropathy is usually made when some patients have serious nerve damage which affects their quality of life. Most previous studies conducted in Asian and European cohorts have explored peripheral neuropathy in rheumatoid arthritis within healthcare systems characterized by earlier diagnosis, standardized longitudinal follow-up, and broader access to advanced disease-modifying therapies. In contrast, patients in sub-Saharan Africa are frequently exposed to prolonged periods of uncontrolled inflammation due to delayed referral, limited availability of specialized care, and heterogeneous therapeutic strategies. By examining peripheral neuropathy in this specific context, our study provides complementary data that extend beyond existing cohorts and highlight how sustained inflammatory burden and disease severity may be associated with nerve involvement. Studies addressing peripheral neuropathies in patients with RA in sub-Saharan Africa are seldom. This contextualized approach contributes to a more global understanding of peripheral neuropathy in rheumatoid arthritis and underscores the need to adapt diagnostic and screening strategies to different healthcare settings. It is in this context that our study aimed to determine the biological and clinical factors in rheumatoid arthritis (rheumatoid factor and activity of the disease) contributing to the presence of peripheral neuropathy in RA.

## 2 Materials and methods

### 2.1 Study design

This was a retrospective study with cross-sectional clinical and biological assessment conducted from February to May 2024, in the rheumatology unit of Douala General Hospital. Patients with rheumatoid arthritis (RA) were recruited according to the ACR (American College of Rheumatology) criteria 1987 (12). Because the selection of patients’ records began in 2004, and these criteria are still employed in our department due to technical limitations, this necessitates reliance on the earlier classification system.

We conducted a survey of the records of patients who came for rheumatology consultations (from 2004 to 2024) to create a database of patients to contact in order to optimize recruitment. Subsequently, we sorted the records to separate those revealing arthritis from other rheumatological diseases. Additionally, records mentioning reactive arthritis, psoriatic arthritis, and rhizomelic pseudopolyarthritis were discarded to retain only the records of patients with rheumatoid arthritis.

All patients with rheumatoid arthritis who gave their consent were included. Patients with RA presenting comorbidities associated with peripheral neuropathy (diabetes mellitus, cancer, hepatitis C, other autoimmune diseases before RA, hypothyroidism, amyloidosis, HIV, sarcoidosis), all hospitalized patients, and all patients taking some neurotoxic drugs (chemotherapeutic agents, infliximab, adalimumab, leflunomide) were excluded.

All selected patients were contacted to participate in this study. Subsequently, we performed a blood sample and administered a questionnaire to collect information on socio-demographic characteristics; DN4 (neuropathic pain in 4 items); DAS-28 (Disease activity score 28); duration of the disease; treatments; other diseases diagnosed after RA to all consenting patients.

Data on anti-citrullinated peptide antibodies (ACPA) and of erythrocyte sedimentation rates were collected from patient records. Patients considered to have peripheral neuropathy were identified based on the diagnosis recorded in their files by the same neurophysiology team. All patients with an abnormal electroneuromyogram (ENMG) associated or not with symptoms and signs in favour of peripheral neuropathy (tactile hypoesthesia, hypoesthesia to pinprick, abolished or diminished osteotendinous reflexes, positive Tinel’s test) were considered as such (13). Information pertaining to the sites, and the characteristics of neuropathies were not evaluated owing to the incompleteness of the data in patient’s files.

A sample size calculation was not performed, as this was an exploratory aiming to assess the presence of peripheral neuropathy in patients’ records solely attributable to rheumatoid arthritis within a single department since its establishment. Moreover, the scope of this exploration was determined by the resources available in the rheumatology unit. A total of 63 patients with RA, divided into two population groups, participated in this study, with 45 participants without PN and 18 participants with PN.

### 2.2 Biological evaluation

The collected blood samples were centrifuged, and then we screened for hepatitis C virus (HCV) by immunochromatography in all patients with peripheral neuropathy using the Rapid HCV kit (Biolab Diagnostic, France). We also evaluated the serum levels of inflammatory markers. The concentrations of CRP and RF were determined by immunoturbidimetry using the BT 1500 analyzer using RF-turbidimetric and CRP-turbidimetric kits (Biotecnica Instruments, Italy).

### 2.3 Evaluation of rheumatoid arthritis disease activity

The disease activity score (DAS-28) of RA was calculated in all participants with the formula using CRP concentrations (14). Patients with a DAS-28 > 5.1 were considered to have severe polyarthritis and patients with a DAS-28 score between 3.2 and 5.1 were considered to have moderate polyarthritis. We collected from the patients’ files the presence of extra-articular manifestations of RA, the duration of the disease, and the type of disease-modifying anti-rheumatic drug (DMARD) used during their disease.

### 2.4 Statistical analysis

SPSS 27 (IBM, United States) software was used for data analysis. Qualitative variables were analyzed using the Chi-square test. The means of RF and ACPA were compared in RA patients with or without PN using the student’s t-test. The significance level was set at *p* ≤ 0.05. A *post hoc* power analysis was performed using G*Power software to estimate the achieved power for detecting a medium effect size (Cohen’s w = 0.3) at an *p* level of 0.05. Considering the total sample size of 63 participants, the calculated statistical power was approximately 0.74, indicating a 74% probability of detecting a moderate association between clinical or biological variables and the occurrence of peripheral neuropathy.

## 3 Results

**Figure 1:**
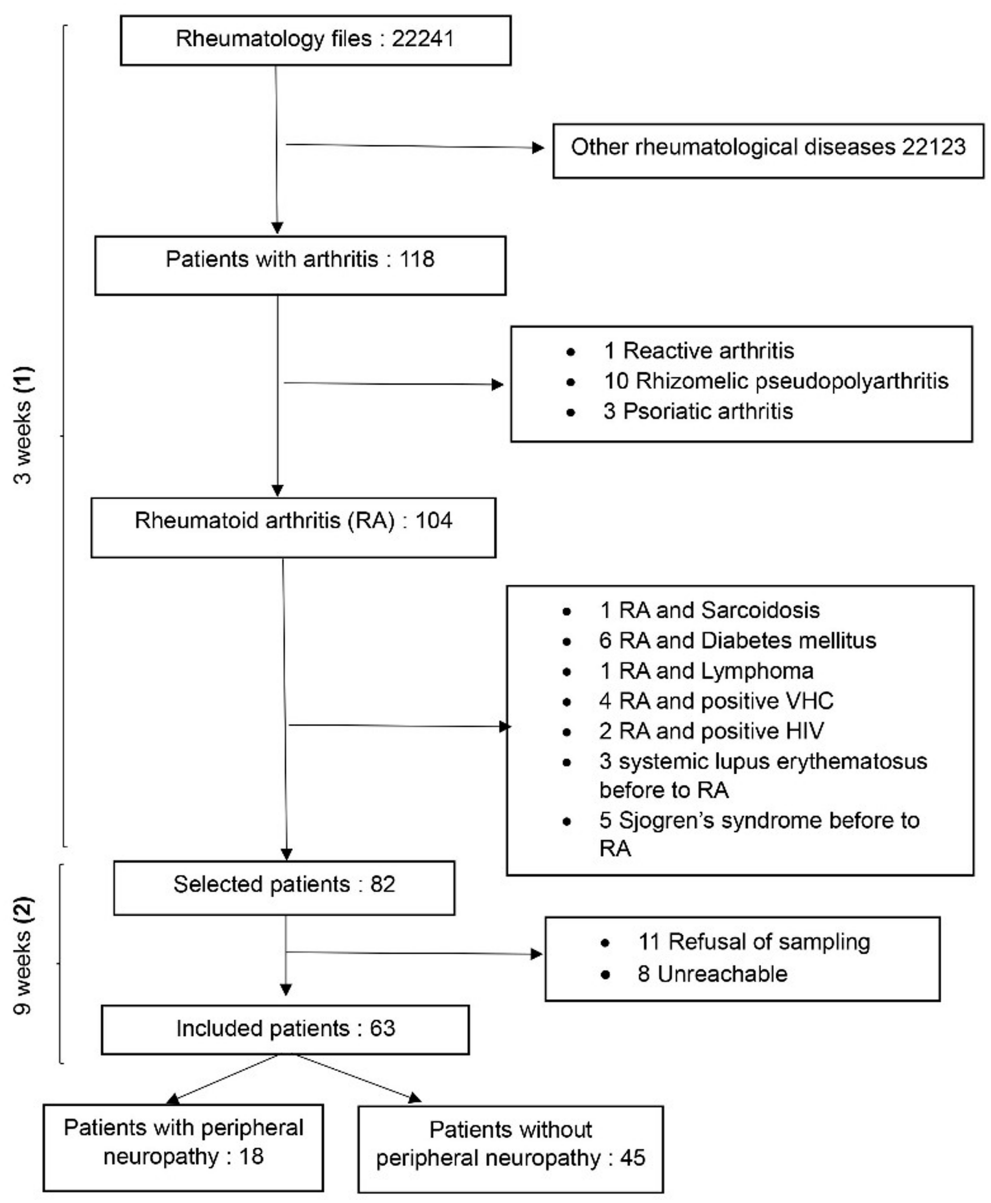
Flow chart of patients recruitment strategy: (1) Census and (2) data collection.

A total of 63 patients participated in the study (Figure 1). The majority (82.5%) were female, and the average age of the population was 51.86 ± 13.077 years, with the most representative age group being [45–54] years (Table 1). Additionally, 71.42% of the population had moderately active RA, while 20.64% had highly active RA. Among the 63 serum samples tested, 71.4% were positive for rheumatoid factor (RF) and 77.8% were positive for anti-citrullinated peptide antibodies (ACPA). RA was seronegative for RF and ACPA in 19.04% of the participants (Table 1).

**Table 1:** Characteristics of the study population.

Variables	Features	Frequency (*N* = 63)	Percentage (%)
Sex	Female	52	82.50
	Male	11	17.50
	<35	7	11.10
	[35–44]	11	17.5
Age group (years)	[45–54]	17	27.00
	[55–64]	16	25.40
	≥65	12	19.00
	DAS-28 ≤ 2.6	1	1.59
	2.6 < DAS-28 ≤ 3.2	4	6.35
DAS-28	3.2 < DAS-28 < 5.1	45	71.42
	DAS-28 > 5.1	13	20.64
RF	RF+	45	71.42
ACPA	ACPA+	45	77.8
FR et ACPA	RF et ACPA-	12	19.04
	Sensory polyneuropathy	1	5.60
	Sensorimotor polyneuropathy	5	27.80
Neuropathy	Multiple mononeuropathy	5	27.80
(*n* = 18)	Tarsal tunnel	2	11.10
	Carpal tunnel	11	38.90

DAS-28, Disease activity score 28; ACPA, Anti-citrullinated peptide antibodies; RF, Rheumatoid factor.

*, Univariate analysis (Raw data); **, Multivariate analysis (Adjusted data); DAS-28, Disease Activity Score 28; DN4, Neuropathic Pain in 4 Questions; CRP, C-Reactive Protein; RF, Rheumatoid Factor; ESR, Erythrocyte Sedimentation Rate; ACPA, Anti-Citrullinated Peptide Antibodies.

Peripheral neuropathy was present in 28.6% of RA cases, distributed as follows: carpal tunnel syndrome (median nerves, sensory and/or motor) 38.9%; sensorimotor polyneuropathy (peroneal nerves, sensory and/or motor; tibial nerves, motor; ulnar nerves, sensory and/or motor; median nerves, sensory and/or motor; sural nerves, sensory) 27.8%; multiple mononeuropathy (ulnar nerves, sensory and/or motor; median nerve, sensory and/or motor; peroneal nerves, sensory and/or motor) 27.8%; tarsal tunnel syndrome (tibial nerve, motor) 11.1%; pure sensory polyneuropathy (superficial peroneal nerves; cutaneous branches of ulnar nerves) 5.6% (Table 1). Among patients with peripheral neuropathy, 72.2% were positive for RF and 77.7% were positive for ACPA (Table 2).

**Table 2:** Factors associated with the presence of peripheral nerve damage in rheumatoid arthritis.

Variables	Neuropathy + *n* = 18 (%)	Neuropathy *n* = 45 (%)	*p*-value	Odds ratio (95% IC)
CRP (mg/L)	51. 19 ± 46.73	32. 24 ± 38.89	0.105	/
ESR (mm/h)	60.41 ± 27.48	40.78 ± 29.86	**0.027**	**8.50 (2.37–36.88)**
ACPA (UI/mL)	178.05 ± 138.39	165.17 ± 170.97	0.766	/
RF +	13 (72.2%)	32 (71.1%)	0.930	/
RF (UI/mL) *	188.33 ± 160.98/	69.44 ± 71.47/2	**0.007**	**39.41 (36.59–201.17)**
RF (UI/mL) **	/	/	**0.017**	**14.85 (1.62–135.79)**
RF > 169.10UI/mL	8 (44.4%)	(4.4%)	**0.003**	**8.20 (2.05–32.75)**
Duration of disease ≥10 years	7 (38.9%)	3 (6.7%)	**0.004**	**8.9 (1.97–40.19)**
DAS-28 > 5.1 *	12 (66.7%)	1 (2.2%)	**<0.001**	**88 (9.64–803.09)**
DAS-28 > 5.1 **	/	/	**<0.001**	**55.13 (5.14–591.04)**
Joint deformities	13 (72.2%)	24 (53.3%)	**0.169**	**/**
Duration of disease, years *	8.44 ± 5.95/	3.97 ± 3.551	**0.006**	**1.21 (2.08–6.94)**
Duration of disease, years **	/	/	0.140	5.57 (0.56–54.74)
DAS-28	5.14 ± 0.536	3.95 ± 0.66	0.615	/
DN4 ≥ 4	5.33 ± 0.84	3.91 ± 1.08	0.622	/
Gout	2 (11.1%)	0 (0.0%)	**<0.001**	/
Corticotherapy	15 (83.3%)	38 (84.4%)	> 0.05	/
Rheumatoid Nodules	2 (11.1%)	3 (6.7%)	0.555	/
Rheumatoid vasculitis	5 (27.8%)	0 (0.0%)	**<0.001**	/
Systemic lupus erythematosus	2 (11.1%)	2 (4.4%)	0.327	/
Sjögren syndrome	3 (16.7%)	4 (8.9%)	0.375	/

The bold values indicate the significant values.

### 3.1 Factors associated with the presence of peripheral neuropathy in rheumatoid arthritis

Rheumatoid vasculitis and gout were significantly associated with the presence of PN in RA (*p* < 0.001). Table 2 interestingly, joint deformities, and rheumatoid nodules were not significantly associated with the presence of PN in RA. Additionally, disease activity score (DAS-28) and the mean age of patients were not significantly associated with PN. Nonetheless, a DAS-28 > 5.1 (severely active rheumatoid arthritis) was significantly associated with the presence of PN in RA (*p* < 0.001, OR = 88). Logistic regression of the two variables (disease duration and DAS-28) indicates that only the rheumatoid arthritis activity score has a significant influence on the presence of PN in the patient.

**Figure 2:**
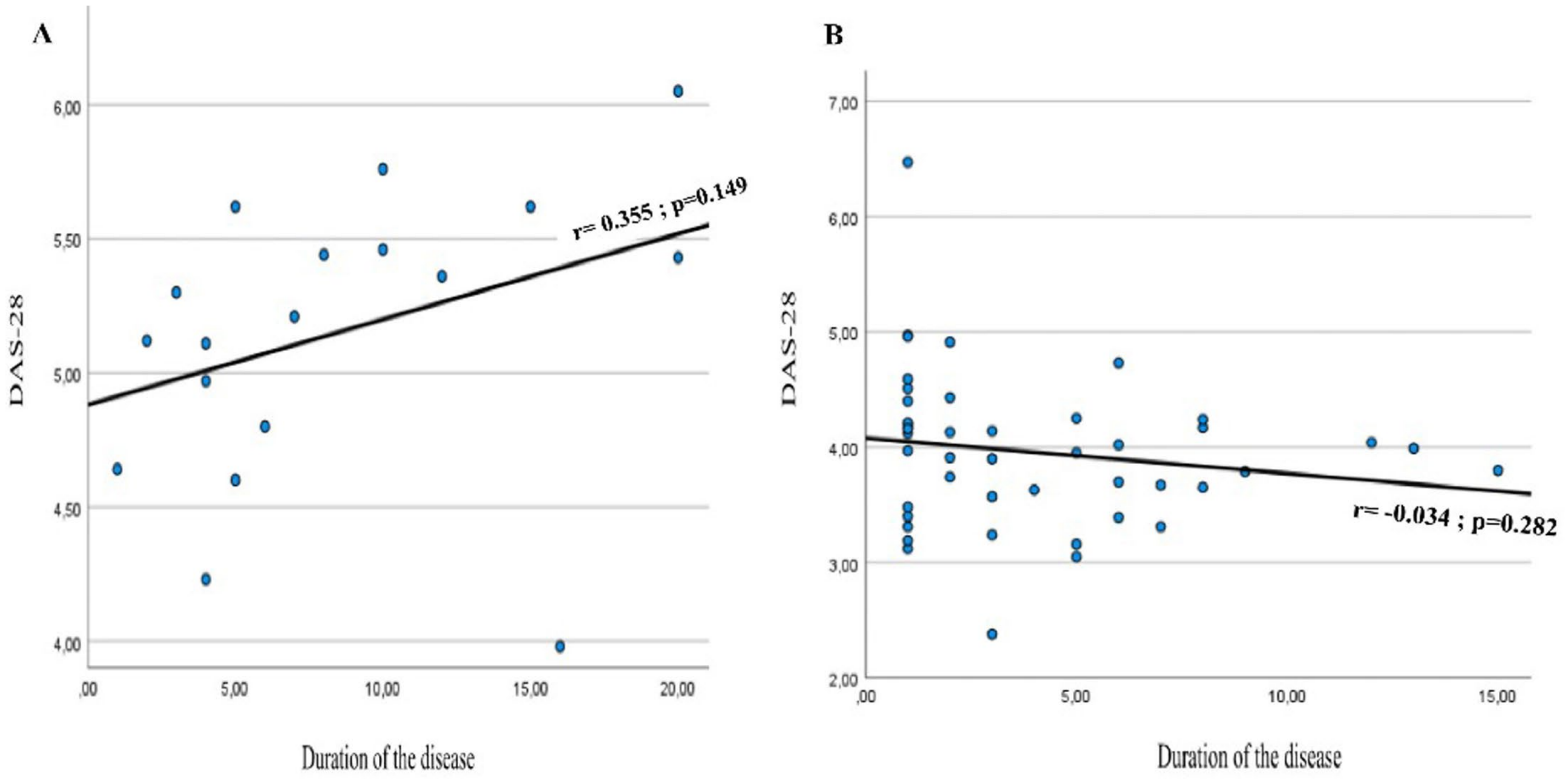
Evolution of the rheumatoid arthritis activity score (DAS-28) according to the duration of the disease (years) in patients with peripheral neuropathy **(A)** and without peripheral neuropathy **(B)**.

There was a positive correlation between the duration of the disease and the rheumatoid arthritis activity score (DAS-28) in patients with PN, though not statistically significant (*r* = 0.355, *p* = 0.149) (Figure 2).

### 3.2 Influence of inflammatory parameters on the presence of peripheral neuropathy in patients with rheumatoid arthritis

The measurement of inflammatory parameters in the serum of patients revealed a significant increase of RF and ESR levels in patients with PN (*p* < 0.001, *p* = 0.029, respectively). The concentrations of CRP and ACPA were also higher in patients with PN, but with no significant difference (*p* = 0.105, *p* = 0.766 respectively) (Table 2).

**Figure 3:**
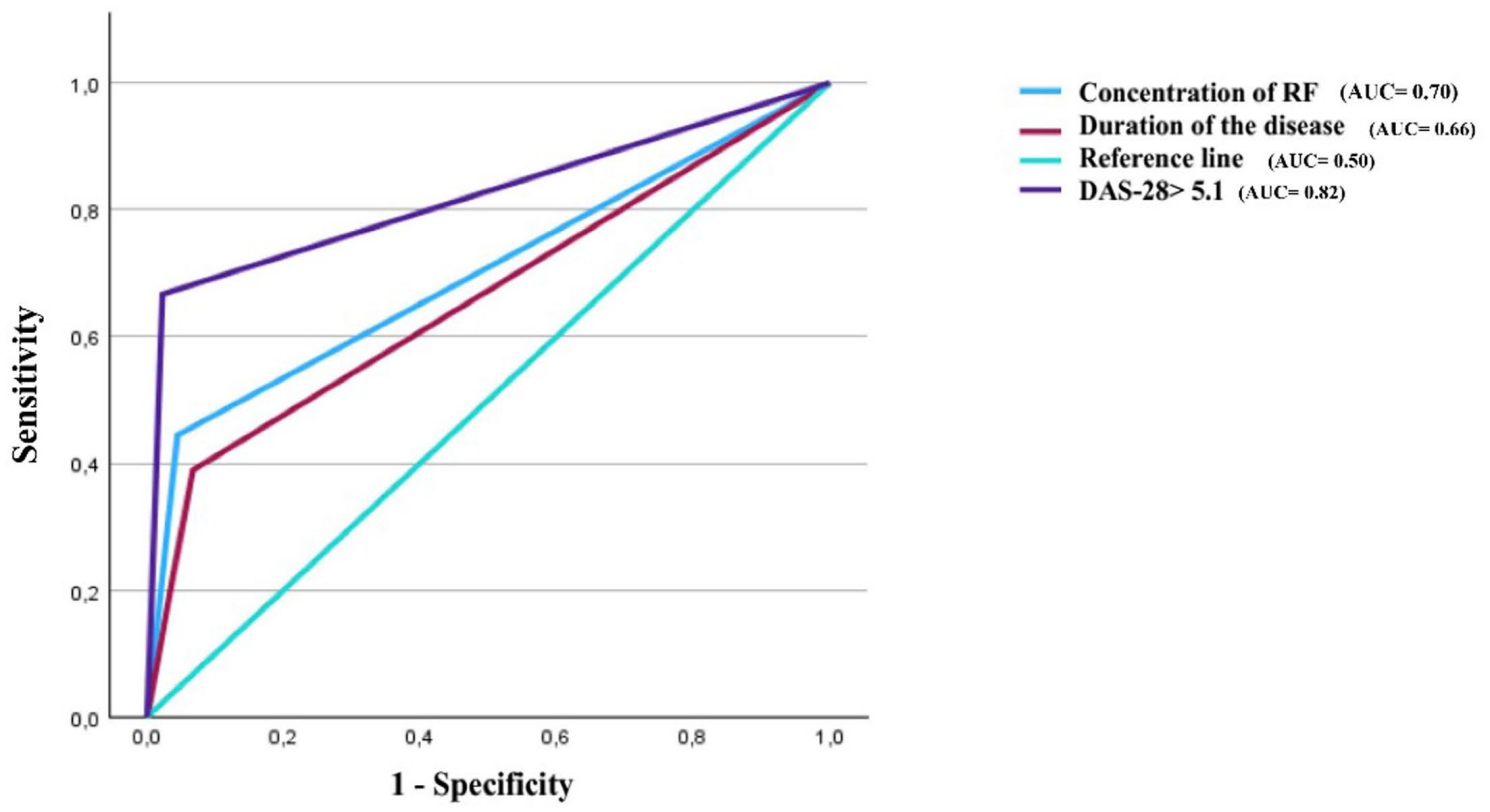
Predictive value of DAS-28 > 5.1, rheumatoid factor concentration, and duration of the disease on the presence of peripheral neuropathy.

The area under the ROC curve for RF concentration was 0.70. This indicates that among two randomly chosen subjects (one with PN and the other without PN), there is a good probability that the subject with PN has a higher serum RF concentration than the subject without PN (Figure 3). When the area under the ROC curve is 0.70 (AUC = 0.70 ± 0.09), the optimal predictive threshold value of RF > 169.10 IU/mL is a factor significantly associated with the presence of PN in RA (*p* = 0.003, OR = 8.20) with a sensitivity of 44.4% and a specificity of 91.1% (Table 2). Additionally, the area under the ROC curve for patients with severely active rheumatoid arthritis (DAS-28 > 5.1) is 0.82 (AUC = 0.82 ± 0.07), our results also show that disease duration is a less efficient model than RF concentration and DAS-28 > 5.1 score (Figure 3). Furthermore, multivariate analysis of these three variables revealed that only RF concentration and severely active rheumatoid arthritis (DAS-28 > 5.1) were significantly associated with the presence of PN (Table 2).

**Figure 4:**
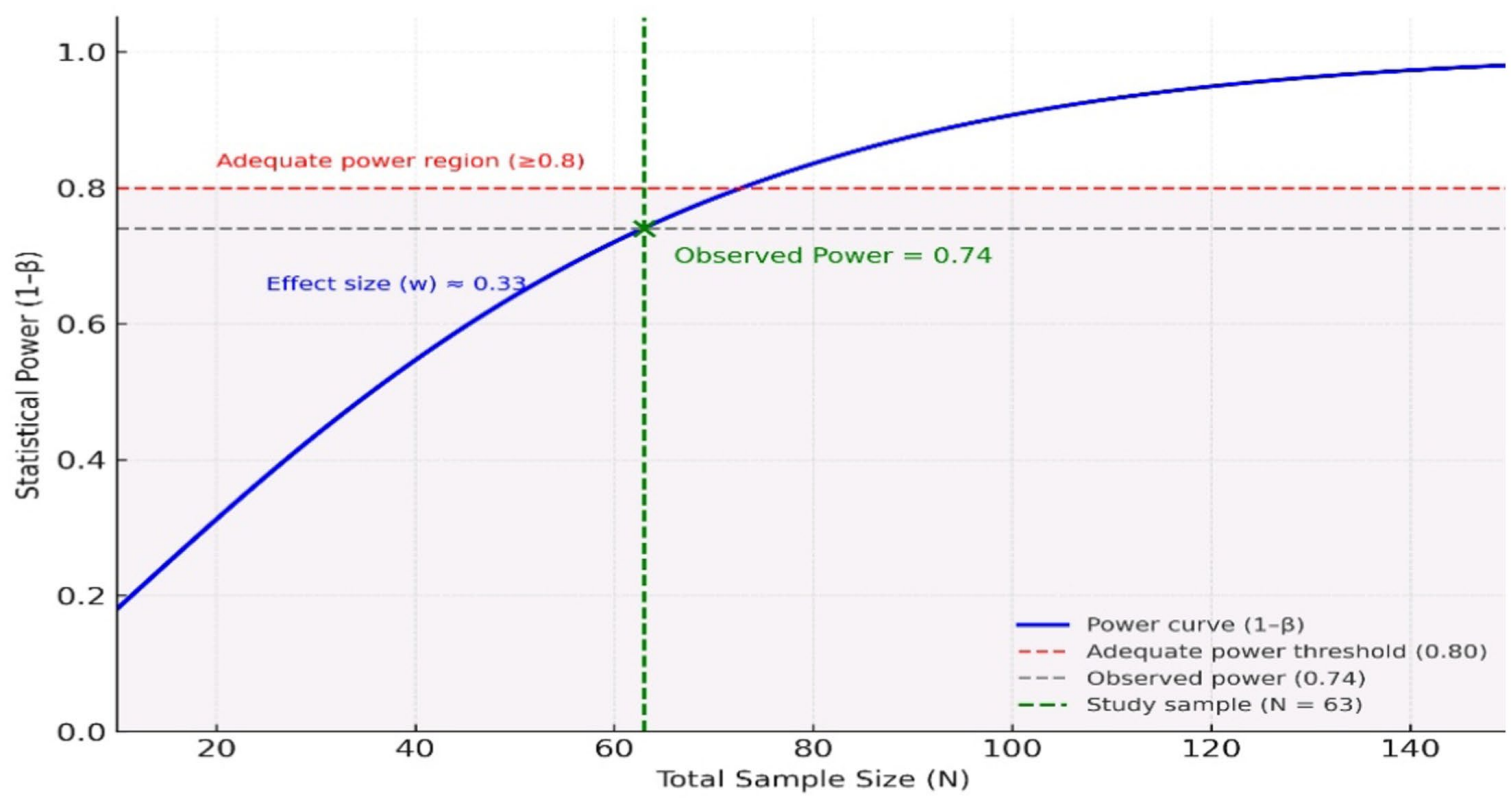
A *post hoc* power analysis to detect a medium effect size.

Since this was an exploratory study, we did not perform *a priori* sample size estimation. However, a *post hoc* power calculation was performed, showing a power of 74% to detect a medium effect size although slightly below the conventional threshold of 80%, this power remains acceptable for an exploratory study and supports the reliability of the main findings (Figure 4).

## 4 Discussion

This study shows that immune dysfunction is a key factor that can contribute to our understanding of the occurrence of peripheral neuropathy in patients with rheumatoid arthritis. These dysfunctions are disease activity (severely active RA) and marked elevation of inflammatory biomarkers (RF, ACPA, CRP, and ESR). These sets of dysregulations can be correlated with neuroinflammation of the nerves leading to the occurrence of peripheral neuropathies.

We found that 82.5% of the population with RA is female, which is similar to the report of Biwas et al. (15) and many other studies on rheumatoid arthritis conducted in Africa (16–18). This might indicate the involvement of female hormonal factors in the onset of rheumatoid arthritis, though the topic continues to generate substantial debate. The average age of the population was 51.86 ± 13.077 years, which is similar to a multicentric study conducted in Algeria by Samy et al. (18).

In our study population, 28.6% of the participants had peripheral neuropathy, a result that is not in line with that of Nidhi et al. (19) and could be explained by the fact that these authors did a prevalence study of PN during which all RA patients recruited underwent examinations for PN, while patients included in our study who were considered to have PN were those with a previously established diagnosis. However, Sim et al. obtained a result similar to our study (20). Carpal tunnel syndrome was the most represented type of neuropathy in RA, which was also shown by Rajeshwari et al. (21).

Among the 63 samples tested, we obtained 71.4% positive cases for RF and 77.8% positive for ACPA. One study in Congo found 70% positive cases for RF and 60% positive for ACPA (16). Another study conducted in Senegal found 58% of positive cases for RF and 78.8% positive for ACPA (17). In India, Agarwal et al. found 80% of cases positive for RF (4). These results are close to what we found in this study. Among patients with PN, 72.2% had a positive RF, a result that is close to that of Agarwal et al. (4).

There was no significant association between the use of corticosteroid therapy and the presence of PN, as also shown by Biwas et al. (15). Additionally, Lanzillo et al. indicate that patients who received corticosteroids have less visible damage on ENMG and even suggest that corticosteroid therapy could have a preventive effect on the progression of peripheral neuropathies (22). Furthermore, there is no significant association between rheumatoid nodules, joint deformities, Sjögren’s syndrome, systemic lupus erythematosus, and the presence of peripheral neuropathy. In contrast, Nidhi et al. (19) found a significant association between the onset of peripheral neuropathy and the presence of rheumatoid nodules. This difference can be explained by the fact that rheumatoid nodules in RA can resolve during the disease when treatment is effective. We note that the development of rheumatoid vasculitis is significantly associated with the presence of PN (*p* < 0.001), which is in agreement with Agarwal et al. (4). This can be explained by the fact that the expansion of pro-inflammatory mediators produced by immune imbalance in RA causing joint inflammation are the same that, in the case of rheumatoid vasculitis, lead to alteration and obstruction of blood vessels causing tissue ischemia of the peripheral nerves irrigated by these vessels. Additionally, gout is a factor significantly associated with the presence of PN in rheumatoid arthritis (23). This can be explained by the fact that colchicine, one of the drugs used in the treatment of gout, can sometimes induce dose-dependent neuropathy, as reported by McEwan et al. (24). Mean age was not significantly different between patients with and without PN, a result similar to that of Yanshan et al. (25).

In agreement with Biwas et al. and Aktekin et al. (15, 26), the duration of RA (years) seems significantly different between the two groups of participants; nonetheless, multivariate analysis of this variable indicates that this parameter could be a confounding factor for the presence of PN in RA. On the other hand, the RA activity score (DAS-28) and DN4 were not significantly different between patients with or without neuropathy, a similar result was found in another study (25). Nonetheless, participants with PN having a DAS-28 > 5.1 have 55 times (OR) more risk of presenting PN than patients with DAS-28 < 5.1 (*p* < 0.001). This result suggests that patients with PN have very high rheumatoid arthritis activity. In patients with peripheral neuropathy, there is a positive correlation (r = 0.355) between RA activity (DAS-28) and RA duration (years). In the sub-Sahara-African context, access to healthcare facilities especially to specialized centers is still limited. Many patients face financial constraints that limit their access to optimal management of RA. Our pharmaceutical market also lacks the most effective anti-inflammatory therapies commonly available in developed countries. According to our findings, we could hypothesize that patients attending tertiary healthcare facilities have a higher risk of peripheral neuropathy. For these reasons, physicians should screen patients with a diagnosis of RA for peripheral neuropathy, as this latter condition increases disease burden to patients with RA. Furthermore, healthcare authorities should include in their program awareness of the general population on RA, and promote continuous medical education for healthcare practitioners on this topic.

In a pathophysiological perspective, the association between severe disease activity in RA and the presence of peripheral neuropathy is addressed. In patients with severe disease activity with a long evolution, chronic and high level of local and systemic inflammation, nerve vasculitis, joints and tissues structural changes around nerves could contribute to nerve injury (22). In patients suffering from peripheral neuropathy, the systemic inflammatory environment could be aggravated by a phenomenon of the body’s acclimatization to treatments despite the progressive increase in therapeutic doses over time, making the body less sensitive to systemic anti-inflammatory molecules. This would cause an elevation of pro-inflammatory agents leading to the progression of arthritis and the increase in disease activity (22).

There was no significant difference in ACPA and CRP concentrations between the two groups. Yanshan et al. (25), found a similar result. However, Kyung et al. found a significant difference (20). More studies are needed to draw a conclusion on this point. We found like Nidhi et al. and Yanshan et al. (19, 25), that the average concentrations of ACPA, CRP and ESR in patients with PN were higher than those in patients without neuropathy, indicating that patients with peripheral neuropathy might present a more altered systemic inflammatory environment than patients without peripheral neuropathy.

We found that the concentration of rheumatoid factor between the two population groups was significantly different (*p* = 0.007). This result is similar to that of Ding et al. (27). The area under the ROC curve for RF concentration in predicting RA complicated by PN is 0.7 (AUC = 0.7), with an optimal threshold value of 169.10 IU/mL. Patients with an RF concentration > 169.10 IU/mL have 8.20 (OR) more risk of presenting peripheral neuropathy than patients with an RF < 169.10 IU/mL. This result is similar to that of Ding et al. (27). This observation could suggest that patients with peripheral neuropathies present a higher disease activity with marked imbalance in pro-inflammatory mediators compared to patients without peripheral neuropathies, leading to greater stimulation of B lymphocytes (plasma cells) producing rheumatoid factor.

The primary objective of this study was to identify the clinical and biological factors associated with the occurrence of peripheral neuropathy in rheumatoid arthritis, rather than to describe the specific electrophysiological or anatomical features of the neuropathies observed. This Findings should be interpreted as associations with overall peripheral neuropathy rather than specific neuropathic subtypes. Nevertheless, the diversity of underlying neuropathic mechanisms, related to systemic inflammation and immune dysregulation, warrants targeted investigation. Further studies, incorporating a larger sample and detailed neurophysiological assessments, are envisaged to better characterize the various neuropathic profiles encountered.

The limitation of our study is the relatively small sample size which prevented us to perform subgroup analysis with different types of peripheral neuropathy. Furthermore, the population with PN is small and disproportionate compared to the population without PN, due to numerous confounding factors that have been ruled out. Nevertheless, the *post hoc* power analysis indicated an acceptable power (74%) for detecting a medium effect size, thereby supporting the robustness of the main associations observed. Moreover, to address cross-sectional design we have used retrospective data on ENMG. The lack of precise chronological tracking for DMARDs treatments limits the assessment of potential cumulative toxicity or dose-dependent effects on peripheral nerves.

## 5 Conclusion

The data presented in this study show that rheumatoid vasculitis, gout, and severe rheumatoid arthritis (DAS-28 > 5.1) are associated with the presence of PN in rheumatoid arthritis. Patients with PN exhibit elevated inflammatory parameters (RF, CRP, ACPA, and ESR), indicating that immune imbalance, one of the causes of peripheral neuropathy, is more significant in patients with PN compared to those without peripheral neuropathy. Despite this was an exploratory study, these findings should be confirmed in larger, prospective studies these data may serve as a guide in the prompt diagnosis of peripheral neuropathy in rheumatoid arthritis.

## Data Availability

The raw data supporting the conclusions of this article will be made available by the authors, without undue reservation.
